# The Mediating Role of Posttraumatic Stress Symptoms in the Relationship between Adult Attachment and Quality of Life

**DOI:** 10.3390/ejihpe14100180

**Published:** 2024-10-07

**Authors:** Gianluca Santoro, Vittorio Lenzo, Alessandro Musetti, Cristiana Caneglias, Lina Rita Crimi, Lucia Sideli, Adriano Schimmenti

**Affiliations:** 1 Department of Humanities, Social Sciences and Cultural Industries, University of Parma, Borgo Carissimi 10, 43121 Parma, Italy; alessandro.musetti@unipr.it; 2 Department of Educational Sciences, University of Catania, Via Biblioteca 4, 95124 Catania, Italy; vittorio.lenzo@unict.it; 3 Department of Human Sciences, LUMSA University, Piazza delle Vaschette 101, 00193 Rome, Italy; c.caneglias@lumsastud.it (C.C.); l.crimi@lumsastud.it (L.R.C.); l.sideli@lumsa.it (L.S.); 4 Department of Human and Social Sciences, UKE—Kore University of Enna, Piazza dell’Università, 94100 Enna, Italy; adriano.schimmenti@unikore.it

**Keywords:** quality of life, attachment, posttraumatic stress, mediation model

## Abstract

There is evidence that anxiety and avoidance toward close relationships (i.e., insecure attachment orientations), as well as posttraumatic stress symptoms (PTSSs), are linked to a poor quality of life. The current study aimed to investigate the potential mediating effects of PTSSs on the associations between insecure attachment orientations and domains of quality of life. A convenience sample of 497 adults (375 females, 75.5%), ranging in age between 18 and 65 years old (M = 32.48, SD = 13.26), was recruited. Participants were administered self-report instruments assessing attachment anxiety and avoidance, PTSSs, and domains of quality of life, including physical health, psychological status, social relationships, and environment. A series of mediation analyses were performed to test the mediating role of PTSSs in the relationships between attachment orientations and domains of quality of life. Results showed that attachment anxiety was related to decreased levels of quality of life in all domains, and that their associations were mediated by PTSSs. Also, attachment avoidance was related to a worse quality of psychological status and social relationships, and PTSSs were a significant mediating variable in these associations. Prevention programs and clinical interventions focused on promoting effective strategies for managing distress might be critical in reducing the impact of distressing events on the quality of life of individuals with insecure attachment.

## 1. Introduction

The World Health Organization conceptualized the quality of life as the “individuals’ perceptions of their position in life in the context of the culture and value systems in which they live and in relation to their goals, expectations, standards and concerns” [1]. Thus, the quality of life encompasses several domains, and some of them, such as physical health, psychological status, social relationships, and features of the surrounding environment, have been found to be highly relevant across cultures [2,3]. 

Previous research suggests that the quality of life is closely related to several facets of individuals’ interpersonal functioning. For example, there is evidence showing that social support is associated with increased quality of life [4,5,6]. Kuczynski and colleagues [7] found that increased social support, social connectedness, emotional intelligence, and intimacy with one’s romantic partner, as well as decreased loneliness and empathetic concern, were associated with heightened levels of quality of life when the effects of other relevant facets of the interpersonal functioning were taken into account in undergraduate students. Also, Kim and colleagues [8] found that decreased participation in various social activities was associated with a poor quality of life in middle-aged adults living alone. Furthermore, stressful life experiences and heightened levels of stress may negatively impact the quality of life [9,10,11,12].

Attachment is a motivational system that underlies an individual’s innate tendency to seek and maintain proximity to caregivers and significant others [13,14]. The attachment theory posits that the quality of early interactions with caregivers is critical in shaping representations of the self, others, and the relationships between the self and others (termed “Internal Working Models”) [15], leading to different attitudes toward both close relationships [16] and responses to stressful events during adulthood [17]. Accordingly, this theory provides a relevant framework for understanding the relationships between individuals’ interpersonal functioning, stress levels in response to adverse or potentially traumatic events, and domains of quality of life. 

### 1.1. Attachment Orientations and Quality of Life

In adulthood, the individual differences in attachment orientations can be identified by examining levels of anxiety and avoidance in close relationships. In more detail, attachment anxiety is related to a negative representation of the self, whereas attachment avoidance is linked with a negative representation of others. Low levels of both attachment anxiety and avoidance denote secure attachment, which is characterized by a representation of the self as lovable and others as trustworthy [18]. Although cultural and contextual factors may have significant effects on the bond with caregivers during childhood and the sense of security in close relationships during adulthood [19,20,21], evidence suggests that the core features of attachment orientations—such as the relevance of the representations of the self and others—do not vary across various cultures [22].

Furthermore, attachment orientations entail different strategies for managing distress. Individuals with secure attachment are prone to appraise threatening events adequately, have confidence in their abilities, and perceive others as available. They also rely on more effective strategies in stressful situations than individuals with anxious or avoidant attachment orientations. Attachment anxiety is linked to the hyperactivation of the attachment system, which may manifest as an overestimation of threats and intense efforts to maintain proximity to others. Conversely, attachment avoidance is related to the deactivation of the attachment system, which involves the minimization of one’s own attachment needs, underestimation of threatening events, and a tendency to rely on oneself [16,23]. The hyperactivation and deactivation of the attachment system may foster difficulties in adequately representing one’s own and others’ mental states and regulating emotions, leading individuals to experience increased levels of distress [24,25].

It is noteworthy that insecure attachment orientations may heighten the risk of impairments in various domains of daily functioning. In fact, the feelings of unworthiness and/or distrust of others embedded in insecure attachment orientations hinder the sense of security in close relationships, increasing the likelihood of exhibiting interpersonal difficulties [18]. For example, research showed that anxious and avoidant attachment orientations are associated with poor sexual and relationship satisfactions [26,27]. Also, there is consistent evidence showing that insecure attachment is associated with mental disorders and the severity of clinical symptoms [28,29], as well as with different aspects of poor mental health, such as loneliness, negative affect, emotion regulation difficulties, poor psychological well-being, and decreased self-esteem [30]. Therefore, insecure attachment, with its related hyperactivation (attachment anxiety) or deactivation (attachment avoidance) of the emotional system, tends to generate interpersonal difficulties and might result in negative psychological outcomes [25,31,32]. Additionally, research showed that insecure attachment is associated with negative physical health conditions [33,34,35]. 

Insecure attachment is linked to a poor quality of life among individuals from the community and those suffering from physical diseases. Brophy and colleagues [36] showed that both attachment anxiety and avoidance predicted decreased levels of quality of life among individuals from the community and that these relationships were partly mediated by self-coldness. Sechi and colleagues [37] examined the relationships between attachment styles, self-esteem, and quality of life in women with a diagnosis of fibromyalgia. Their findings showed that all domains of quality of life (including physical health, psychological status, social relationships, and environment) were positively associated with secure attachment (characterized by low attachment anxiety and avoidance) and negatively associated with insecure attachment styles, including dismissing (characterized by high attachment avoidance and low attachment anxiety), preoccupied (characterized by high attachment anxiety and low attachment avoidance), and fearful (characterized by high attachment anxiety and avoidance) attachment. Also, they found that self-esteem significantly mediated the relationships between attachment styles and quality of life. Pistorio and colleagues [38] found that, among patients with psoriasis, the need for approval—that is, a core characteristic of attachment anxiety—predicted an increased severity of skin disease effect on quality of life. Furthermore, a recent systematic review showed that both attachment anxiety and avoidance are related to poor quality of life among early-stage breast cancer patients [39]. Although there is consistent evidence that insecure attachment is related to impairments in individuals’ functioning and worse quality of life, further research is needed to elucidate the role of responses to distressing or potentially traumatic events in the relationships between insecure attachment orientations and quality of life. 

### 1.2. Posttraumatic Stress Symptoms and Quality of Life

The direct or indirect exposure to traumatic events might lead to developing various posttraumatic stress symptoms (PTSSs)—which are core features of posttraumatic stress disorder (PTSD)—including intrusive symptoms (e.g., flashbacks or nightmares) related to past traumatic events, avoidance of inner or external cues of past traumatic experiences (e.g., places or people), negative alterations in cognitions and mood (e.g., feelings of helplessness), and alterations in arousal and reactivity, as well as distress or impairments in several areas of daily functioning [40]. Lifetime prevalence rates of PTSD are higher in high-income countries compared to upper-middle and low-lower middle-income countries [41]. Also, certain cultural factors, including stigma, acculturation, and discrimination, may increase the risk of developing PTSD and hinder treatment-seeking behaviors [42].

A meta-analysis revealed that individuals with PTSD exhibited high levels of impairment in several domains of daily functioning, such as abilities related to general tasks and demands, mobility, self-care, interpersonal interactions and relationships, and social and community life, among others [43]. The PTSSs are not only associated with negative outcomes in individuals’ functioning but also with worse physical health conditions [44,45].

Furthermore, previous research showed that PTSSs are related to decreased levels of quality of life among individuals who were exposed to potentially traumatic events. For example, Nygaard and Heir [46] examined the relationships between these variables in adults who survived a tsunami. Their findings revealed that PTSSs six months after the disaster were negatively related to the quality of life two years after the disaster and that the quality of life six months after the disaster was negatively related to PTSSs two years after the disaster. Giacco and colleagues [47] found a negative association between hyperarousal and quality of life at one-year follow-up, as well as a temporal reversal association between these variables, in individuals who were exposed to war-related potentially traumatic events. Huijts and colleagues [48] showed that PTSSs predicted a worse quality of life in refugees who were exposed to man-made traumatic events.

### 1.3. The Role of Posttraumatic Stress Symptoms in the Relationships between Attachment Orientations and Quality of Life

There is consistent evidence suggesting that both anxious and avoidant attachment orientations may contribute to the heightened severity of PTSSs [49]. For instance, previous meta-analytic findings showed that PTSSs are negatively associated with secure attachment and positively associated with both attachment anxiety and attachment avoidance [50]. The negative representation of the self and/or others underlying insecure attachment orientations undermines feelings of security and can lead to maladaptive strategies for dealing with potentially traumatic events. More specifically, the hyperactivation of the attachment system might increase the likelihood of experiencing intrusive thoughts and memories related to past traumatic experiences, whereas the deactivation of the attachment system might involve the denial of painful emotional states and avoidance of stimuli related to traumatic events [17]. Although the deactivation of the attachment system might prevent conscious exposure to painful emotional states related to traumatic events [14,16], persistent stressful conditions might overcome the individual’s capacity to tolerate distress, resulting in negative psychological outcomes [24,25]. Thus, individuals who resort to hyperactivation or deactivation of the attachment system might be highly prone to develop PTSSs when faced with distressing or otherwise potentially traumatic events, experiencing impairments in various domains of their daily functioning, such as physical health, psychological status, social relationships, as well as difficulties in adjusting to their environment. Accordingly, PTSSs might have a critical role in worsening the quality of life among individuals with insecure attachment orientations. 

### 1.4. Aims of the Study

**Figure 1:**
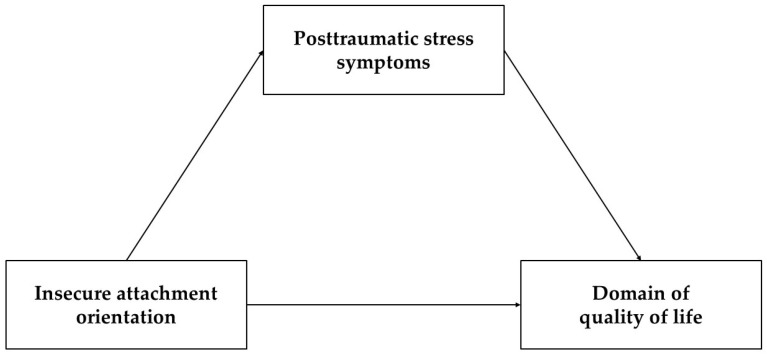
Model depicting the potential mediating role of posttraumatic stress symptoms in the relationships between insecure attachment orientations and domains of quality of life.

As reviewed above, attachment orientations, PTSSs, and quality of life are interconnected to each other. However, the potential mediating role of PTSSs in the relationship between attachment insecurities and quality of life has yet to be examined. Investigating the role of attachment orientations and PTSSs across different domains of quality of life might provide relevant insights for prevention programs and clinical interventions. Therefore, the present study tested the following hypotheses:

1. Attachment anxiety and avoidance are associated with increased levels of PTSSs;
2. Attachment anxiety and avoidance are associated with a poor quality of physical health, psychological status, social relationships, and environment;
3. PTSSs are associated with a poor quality of physical health, psychological status, social relationships, and environment;
4. Attachment anxiety and avoidance are indirectly associated with various domains of quality of life, including physical health, psychological status, social relationships, and environment, through the mediating effects of PTSSs (the hypothesized model is displayed in Figure 1).

It is noteworthy that the core features of insecure attachment orientations are invariant across cultural contexts and that the quality of physical health, psychological status, social relationships, and environment are highly relevant to individuals’ functioning in several cultures. In light of these considerations, we recruited a sample of participants from the community in order to deepen the understanding of the potential pathways (i.e., the effects of PTSSs) that underlie the relationships between attachment orientations and various domains of quality of life.

## 2. Materials and Methods

### 2.1. Participants and Procedures

The sample of the current study comprised 497 adults from the community (375 females, 75.5%), who were aged between 18 and 65 years old (M = 32.48, SD = 13.26). Male and female participants did not differ with respect to age (see Table 1). About half of the participants had a Bachelor’s degree or higher (*n* = 260, 52.3%), whereas 217 had a high school diploma (43.7%), and 27 had a primary school certificate or lower secondary school diploma (4%). There were 454 workers and/or student (91.5%) and 43 unemployed or retired (8.5%). Among participants, 308 were not married (62%), 164 were married or lived with their partner (33%), 22 were separated or divorced (4.4%), and 3 were widowers or widows (0.6%). The large majority of participants lived with other persons in their household (*n* = 447, 89.9%).

Participants were recruited through flyers affixed in public places (e.g., gyms, pubs, university spaces, and hair salons) and advertisements posted on social media platforms (e.g., WhatsApp and Instagram). Each flyer and advertisement presented a QR code and a link that allowed people interested in the study to access an anonymous online survey. Also, flyers and advertisements included a request to share the link and QR code with other people. The online survey contained an informed consent schedule, a sociodemographic schedule, and self-report instruments. The sociodemographic schedule and self-report instruments were automatically administered only to people who submitted their agreement to participate in the study on the informed consent schedule. No time restriction was set for completing the sociodemographic schedule and self-report instruments, and no data on compilation time was collected. The estimated time to complete the online survey was approximately 20 min.

Participants were recruited based on the following eligibility criteria: (a) being between 18 and 65 years old; (b) not having suffered or actually suffering any physical illness that hinders the engagement in common daily activities (e.g., neurological disorders); and (c) not having a self-reported history or current diagnosis of serious mental disorders, such as bipolar, psychotic disorders or dementia. The second and third eligibility criteria were evaluated through ad hoc dichotomous questions. Participants received no monetary or other forms of compensation, and their involvement in the study was entirely voluntary.

The current study was carried out in accordance with the Helsinki Declaration and approved by the IRB of the University of Catania (protocol code: Ierb-Edunict-2023.01.16/5). All data were collected between January and December 2023.

### 2.2. Measures

The *World Health Organization Quality of Life BREF* (WHOQOL-BREF [2], Italian validation [51]) is a self-report instrument that evaluates four domains of quality of life, including physical health, psychological status, social relationships, and environment. The WHOQOL-BREF includes 26 items, and each item is rated on a 5-point Likert scale (e.g., 1 = “Not at all”; 5 = “Completely”). The following question is an example of an item: “To what extent do you have the opportunity for leisure activities?” (evaluating the environment domain). Scores on each domain scale were computed by averaging scores on the items and multiplying the result by 4. Each domain scale has a potential score range of 4 to 20, with higher scores indicating a higher quality of life. Previous research showed that the WHOQOL-BREF has good psychometric properties, such as validity and reliability [2,52]. In the current study, Cronbach’s alpha was 0.75 for the physical health scale (7 items), 0.80 for the psychological status scale (6 items), 0.64 for the social relationships scale (3 items), and 0.70 for the environment scale (8 items).

The *Relationship Questionnaire* (RQ [18], Italian adaptation [53]) is a self-report instrument assessing four prototypical attachment styles based on the positive or negative representation of the self and others. The secure attachment style is characterized by a positive representation of both self and others; the dismissing attachment style is characterized by a positive representation of the self and a negative representation of others; the preoccupied attachment style is characterized by a negative representation of the self and a positive representation of others; and the fearful attachment style is characterized by a negative representation of both self and others. The RQ consists of four first-person statements, that are rated on a 7-point Likert scale (1 = “Strongly disagree”; 7 = “Strongly agree”). Thus, each attachment style is evaluated through one first-person statement. The following statement is an example of an item: “I am somewhat uncomfortable getting close to others. I want emotionally close relationships, but I find it difficult to trust others completely, or to depend on them. I sometimes worry that I will be hurt if I allow myself to become too close to others” (referring to a fearful attachment style). In line with Bartholomew and Horowitz’s theoretical framework [18], we computed scores on two scales evaluating attachment anxiety [(scores on fearful attachment style scale + scores on preoccupied attachment style scale) − (scores on secure attachment style scale + scores on dismissing attachment style scale)] and attachment avoidance [(scores on fearful attachment style scale + scores on dismissing attachment style scale) − (scores on secure attachment style scale + scores on preoccupied attachment style scale)], respectively. Scores on attachment anxiety and avoidance can range from −12 to +12, with higher scores indicating higher levels of insecure attachment orientations. The psychometric properties of RQ included discriminant validity and test-retest reliability [54,55].

The *Impact of Event Scale—Revised* (IES-R [56], Italian validation [57]) is a self-report instrument assessing the severity of distress related to past traumatic events. The IES-R consists of 22 items rated on a 5-point Likert scale (0 = “Not at all”; 4 = “Extremely”). The following statement is an example of an item: “Reminders of it caused me to have physical reactions, such as sweating, trouble breathing, nausea, or a pounding heart”. Scores on a total scale were computed by summing scores on all items. Scores on the total scale can vary from 0 to 88. Higher scores indicate higher levels of PTSSs. The IES-R demonstrated good psychometric properties, such as concurrent validity and discriminant ability for PTSD [58,59]. In the current study, Cronbach’s alpha was 0.94 for the IES-R total scale.

A sociodemographic schedule was administered to collect information about sex, age, marital status, and living arrangements.

### 2.3. Statistical Analyses

As the degree of association between attachment and quality of life varied across previous studies due to the recruitment of samples with different characteristics and the use of various instruments, we estimated the minimum sample size using a conservative approach. Both Type I and Type II error rates were set at 0.05, and the expected correlation coefficient was set at *r* = 0.20. The required sample size was estimated to be *n* = 319. Then, we computed preliminary analyses. Thus, descriptive statistics were performed for all the investigated variables, and sex differences in insecure attachment orientations, PTSSs, and domains of quality of life were examined through a series of t-tests. 

The associations between age, anxious and avoidant attachment orientations, PTSSs, and domains of quality of life were investigated through Pearson’s *r* correlation coefficients. Four multiple linear regression models were computed to examine the effects of sociodemographic characteristics (i.e., sex and age), anxious and avoidant attachment orientations, and PTSSs on each domain of quality of life: each model included, respectively, the perceived quality of physical health, psychological status, social relationships, and environment as dependent variables. 

The potential mediating role of PTSSs in the relationships between insecure attachment orientations and domains of quality of life was investigated through eight mediation analyses. Each mediation model included attachment anxiety or attachment avoidance as an independent variable, PTSSs as a mediating variable, and a single domain of the quality of life as a dependent variable. Sociodemographic characteristics and the contrasting insecure attachment orientation scores (e.g., attachment avoidance scores for models with attachment anxiety as a predictor) were included as covariates in mediation models. Scores on independent and mediator variables were mean-centered to reduce collinearity. As the t-tests revealed that there were significant sex differences in certain variables of interest, we further tested our results showing statistically significant mediating effects of PTSSs by taking into account the potential interactions between insecure attachment orientations and sex on both PTSSs and domains of quality of life. Thus, we performed moderated mediation analyses. The investigated models included a single insecure attachment orientation as an independent variable, PTSSs as a mediating variable, and a single domain of quality of life as a dependent variable, as well as age and the contrasting insecure attachment orientation as covariates. Scores of independent variables and the moderator variable were mean-centered. Five thousand percentile bootstrap samples were computed to examine the significance of indirect effects in mediation and moderated mediation models. A *p*-value of 0.05 was set as the critical level for statistical significance: if the 95% confidence interval does not include the value of 0, the indirect effect is statistically significant. Mediation and moderated mediation models were tested using Process Macro version 4.1 for SPSS [60].

## 3. Results

### 3.1. Descriptive Statistics and Sex Differences

Descriptive statistics are reported in Table 1. Mean and standard deviation scores on RQ scales indicate that anxious and avoidant attachment orientations were heterogeneously distributed among participants. The mean score on the IES-R total scale was higher than the suggested cutoff value of 0.33 [58], suggesting that participants may exhibit clinically relevant levels of PTSSs. It is noteworthy that data were collected at the final stages and immediately after the public health emergency of international concern related to the COVID-19 pandemic, which might have evoked intense feelings of fear among participants [61] and have represented a potentially traumatic event. Mean scores on the WHOQOL-BREF scales were comprised in the range for an acceptable quality of life [52]. Data for age, RQ, IES-R, and WHOQOL-BREF scales were normally distributed among participants, as the absolute values of kurtosis and skewness were lower than 7 and 2, respectively, in a sample of more than 300 participants [62].

Sex differences for the variables of interest are reported in Table 2. Females reported higher scores on the IES-R total scale evaluating PTSSs (*d* = 0.39) and lower scores on WHOQOL-BREF scales evaluating physical (*d* = 0.21), psychological (*d* = 0.25), and environmental (*d* = 0.37) domains of the quality of life.

### 3.2. Correlation Analyses

Pearson’s *r* correlation coefficients for the associations between the investigated variables are reported in Table 3. Both anxious and avoidant attachment orientations were positively associated with PTSSs. Also, both insecure attachment attitudes and PTSSs were negatively associated with all domains of quality of life (i.e., physical health, psychological status, social relationships, and environment). Age was negatively associated with anxious attachment attitudes and PTSSs and positively associated with the quality of psychological status.

### 3.3. Multiple Linear Regression Analyses

The results of the multiple linear regression analyses are reported in Table 4. Attachment anxiety and PTSSs were significant predictors of decreased levels of all domains of quality of life (i.e., physical health, psychological status, social relationships, and environment). Also, attachment avoidance significantly predicted a worse quality of social relationships. Being female predicted increased levels of the quality of social relationships, whereas being male predicted increased levels of the quality of the environment. Age was a significant predictor of decreased levels of the quality of both social relationships and the environment. 

### 3.4. Mediation and Moderated Mediation Analyses

**Figure 2:**
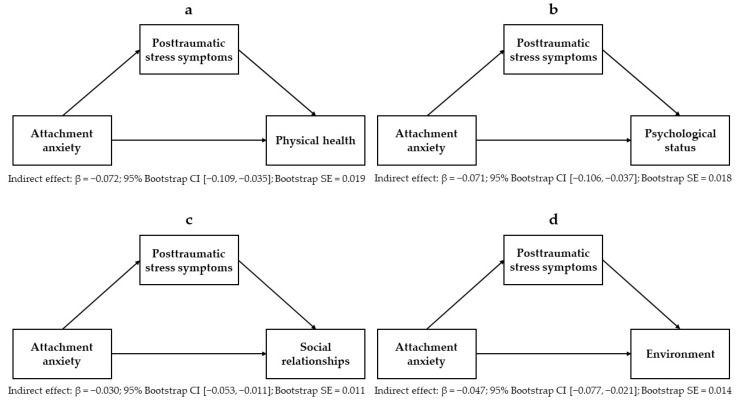
Mediating effects of posttraumatic stress symptoms on the relationships between attachment anxiety and the quality of physical health (**a**), psychological status (**b**), social relationships (**c**), and environment (**d**).

**Figure 3:**
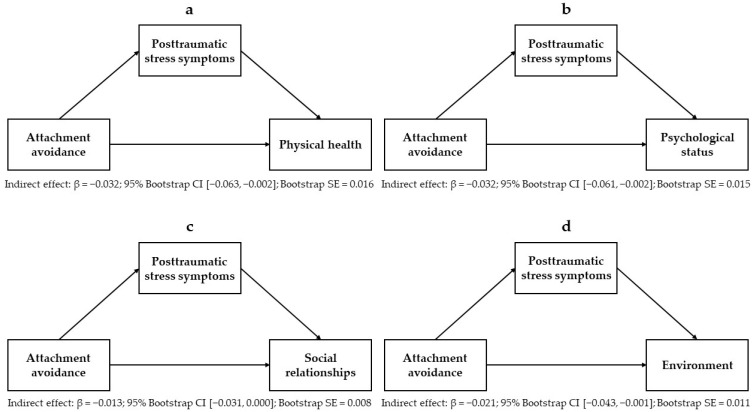
Mediating effects of posttraumatic stress symptoms on the relationships between attachment avoidance and the quality of physical health (**a**), psychological status (**b**), social relationships (**c**), and environment (**d**).

Mediation models encompassing the relationships between attachment anxiety, PTSSs, and each domain of quality of life are displayed in Figure 2a–d. Estimates for each mediation model, including attachment anxiety as an independent variable, are reported in Table 5. Mediation models encompassing the relationships between attachment avoidance, PTSSs, and each domain of quality of life are displayed in Figure 3a–d. Estimates for each mediation model, including attachment avoidance as an independent variable, are reported in Table 6. Results showed that the negative associations between attachment anxiety and all domains of quality of life (i.e., physical health, psychological status, social relationships, and environment) were partially mediated by PTSSs. Also, PTSSs had full mediating effects on the negative association between attachment avoidance and the quality of psychological status and partial mediating effects on the negative association between attachment avoidance and the quality of social relationships. Sociodemographic variables were significant covariates in the mediation models, including attachment anxiety and avoidance as independent variables and the quality of social relationships (being female: *B* = 0.797, 95% CI [0.210, 1.383], (se = 0.299), *p* = 0.008, β = 0.107; age: *B* = −0.026, 95% CI [−0.046, −0.007], (se = 0.010), *p* = 0.008, β = −0.110) and environment (being male: *B* = −0.674, 95% CI [−1.116, −0.232], (se = 0.225), *p* = 0.003, β = −0.130; age: *B* = −0.015, 95% CI [−0.030, 0.000], (se = 0.0007), *p* = 0.043, β = −0.089) as dependent variables. Also, the contrasting attachment orientations (attachment anxiety scores for avoidance models, and attachment avoidance scores for anxious models) were significant covariates in the mediation models.

Moderated mediation analyses revealed that the interaction between attachment anxiety and sex had significant predicting effects on the quality of social relationships (*p* = 0.040, 95% CI [0.006, 0.251]); in contrast, the interaction between attachment anxiety and sex was a not significant predictor of PTSSs and the quality of physical health, psychological status, and environment. Also, the interaction between attachment avoidance and sex had no significant effects on PTSSs and the quality of psychological status and social relationships. Although we found a significant interaction between attachment anxiety and sex in predicting the quality of social relationships, moderated mediation analyses showed no significant differences between conditional indirect effects on the quality of physical health (95% Bootstrap CI [−0.056, 0.025]), psychological status (95% Bootstrap CI [−0.061, 0.026]), social relationships (95% Bootstrap CI [−0.033, 0.014]) and environment (95% Bootstrap CI [−0.035, 0.016]) in models encompassing the interaction between attachment anxiety and sex, and no significant differences between conditional indirect effects on psychological status (95% Bootstrap CI [−0.086, 0.020]) and social relationships (95% Bootstrap CI [−0.046, 0.012]) in models encompassing the interaction between attachment avoidance and sex.

## 4. Discussion

The current study aimed to test the potential mediating effects of PTSSs on the relationships between insecure attachment orientations (i.e., attachment anxiety and avoidance) and various domains of the quality of life (i.e., physical health, psychological status, social relationships, and environment). We observed significant sex differences in the variables of interest among participants. In line with previous research, females reported higher levels of PTSSs [63] than males. Additionally, females reported the lowest levels of the perceived quality of physical health, psychological status, and environment. These findings are partially consistent with previous research showing that females may report a higher quality of social relationships but a lower quality of psychological status compared to males [52]. However, previous research suggests that significant sex differences might be observed in specific degrees of quality of life [64]. A younger age was associated with increased attachment anxiety and PTSSs, supporting previous studies [41,65]. Additionally, older age was associated with an increased perceived quality of psychological status. This finding aligns with previous research showing that an older age is associated with increased levels of psychological well-being [66].

Correlation analyses showed significant associations between insecure attachment orientations, PTSSs, and all domains of quality of life. In line with our first hypothesis, both attachment anxiety and avoidance were positively associated with increased levels of PTSSs. This finding further supports previous research suggesting that individuals with insecure attachment orientations may display specific difficulties in processing and regulating painful emotional states related to past stressful (or otherwise potentially traumatic) events, increasing the risk of developing PTSSs [49,50]. Both anxious and avoidant attachment orientations were also negatively correlated to all domains of quality of life. These findings support our second hypothesis. In accordance with previous literature, individuals with insecure attachment orientations might exhibit interpersonal difficulties [18,26,27], poor physical health [34,35], and worse psychological status [31], due to their negative representations of the self and/or others and their tendency to rely on potentially maladaptive strategies related to the hyperactivation and deactivation of the attachment system. These characteristics of insecure attachment orientations might worsen the adjustment of individuals in various daily life contexts, decreasing the perceived quality of various facets of the surrounding environment, such as security, home conditions, financial resources, and opportunities to engage in leisure activities, among others. It is noteworthy that correlations (from weak to moderate correlation coefficients) between attachment anxiety and domains of quality of life were stronger compared to correlations between attachment avoidance and domains of quality of life (very weak but significant correlation coefficients), suggesting that individuals with anxious attachment might experience a worse quality of life compared to individuals with avoidant attachment. In line with the attachment theory, the deactivation of the attachment system might hinder the conscious experience of painful emotional states and one’s own attachment needs [14,16], promoting a less negative perception of one’s own difficulties and conditions. 

Additionally, PTSSs negatively correlated with all domains of quality of life, in accordance with our third hypothesis. This finding is consistent with research showing that individuals with PTSD might exhibit a worse quality of life [47,67], as well as impairments in several areas of daily functioning [43] and negative health outcomes [44,45]. 

Regression analyses showed that attachment anxiety was associated with a worse quality of physical health, psychological status, social relationships, and environment, and that attachment avoidance was associated with a worse quality of social relationships. Additionally, PTSSs were negatively related to domains of quality of life in all regression models. Mediation analyses provide critical insight to extend the understanding of the relationships between insecure attachment orientations, PTSSs, and domains of quality of life. Results showed that PTSSs were a significant mediating variable in the relationships between attachment anxiety and all domains of quality of life, and between avoidant attachment and the quality of psychological status and social relationships. These findings partially supported our fourth hypothesis, as we found no significant mediating effects of PTSSs on the associations between attachment avoidance and the quality of physical health and environment. Specifically, PTSSs had partial mediating effects on the associations between attachment anxiety and domains of quality of life. Thus, individuals with anxious attachment might exhibit poor daily functioning when exposed to stressful life events. In fact, the sense of elevated vulnerability embedded in the negative representation of the self, the high preoccupation with being rejected by others [18], and the tendency to rely on the hyperactivation of the attachment system [23] might heighten vulnerability to PTSSs, which might further increase the tendency to intensify one’s own signals of distress, proximity-seeking behaviors, and feelings of hopelessness. Thus, PTSSs might contribute to greater impairments in various contexts of daily functioning among individuals with anxious attachment. Additionally, PTSSs were a full mediator variable in the negative association between attachment avoidance and the perceived quality of psychological status, and a partial mediator variable in the negative association between attachment avoidance and the perceived quality of social relationships. Thus, PTSSs might have a critical role in explaining the poor perceived quality of psychological status and social relationships among individuals with avoidant attachment. In fact, the deactivation of the attachment system might lead individuals to intensify their tendency to rely on themselves and avoid cues related to stressful (or potentially traumatic) events [17,23]. These attachment-related strategies might hinder self- and inter-regulation of emotional states, fostering distress and worsening the perceived quality of one’s own well-being. Accordingly, a worse perceived quality of psychological status among individuals with avoidant attachment might indicate a breakdown of their capacities to tolerate painful emotional states related to past potentially traumatic events. Also, our findings suggest that PTSSs might have a relevant role in the negative perception of social relationships among individuals with avoidant attachment. From an attachment perspective, PTSSs might further increase the tendency to dismiss intimacy with others to maintain the deactivation of the attachment system [14] or, in enduring stressful conditions, to experience intense feelings of insecurity in close relationships due to the representation of others as unavailable and untrustworthy underlying attachment avoidance [18].

Although we found no sex differences in the quality of social relationships through the t-test, mediation analyses showed that being female was associated with an increased quality of social relationships when its effects were controlled for age, attachment orientations, and PTSSs. This finding aligns with previous research suggesting that females may exhibit a better quality of social relationships than males [52]. Furthermore, the t-test revealed significant sex differences in PTSSs and domains of quality of life. However, moderated mediation analyses showed that the mediating effects of PTSSs in the associations between insecure attachment orientations and domains of quality of life were not conditioned by sex. As previous research suggests that females might exhibit higher levels of PTSSs than males due to higher levels of certain psychological factors, such as negative peritraumatic emotional responses and post-traumatic negative cognitions about self and the world [68], further studies are needed to investigate whether these psychological factors are involved in the relationships between insecure attachment orientations, PTSSs and quality of life. 

The current study involves some limitations, which should be addressed to improve the methodological design of future research. Firstly, the sample consisted of participants from the community, hindering the generalizability of our findings to individuals with physical diseases or mental disorders. Thus, future research might investigate the relationship between insecure attachment orientations, PTSSs, and domains of quality of life among clinical samples. Additionally, we employed a snowball sampling procedure based on the distribution of flyers and advertisements on social media platforms. This may enhance biases in the sampling process, as participants might exhibit specific characteristics that were not evaluated in the current study (e.g., socioeconomic status, culture, and so on). Thus, our findings may not be directly extended to any group from the community. For example, most of the participants were females (75.5%). Although the effects of sex on the relationships between attachment orientations, PTSSs, and domains of quality of life were accounted for in the mediation analyses, the high percentage of females in the sample might have potentially affected our results. Furthermore, participants reported high scores on the IES-R scale evaluating PTSSs (mean scores above the suggested cut-off value of 0.33). Future studies might recruit participants through sampling procedures other than snowball sampling in order to balance the ratio for different sociodemographic variables, such as sex, and further test our hypotheses on participants who do not report clinically relevant scores on scales evaluating PTSSs. Additionally, participants were administered self-report instruments, which might heighten the risk for bias. Especially, the WHOQOL-BREF scales demonstrated from not satisfactory to acceptable internal consistency in the current study, as Cronbach’s alpha values were comprised from 0.64 (for the quality of social relationships) to 0.80 (for the quality of psychological status). It is noteworthy that these findings align with previous research on the Italian population [51,52] and that Cronbach’s alpha is sensitive to the number of items (the WHOQOL-BREF scale evaluating the quality of social relationships includes three items). Future studies might adopt structured or semi-structured interviews to evaluate the variables of interest. Finally, the cross-sectional design of the study did not allow us to ascertain the causal relationships between attachment orientations, PTSSs, and domains of quality of life. For example, clinically relevant levels of PTSSs might undermine the sense of security in close relationships, fostering insecure attachment orientations [69]. Thus, further research based on longitudinal design might be critical to detect the directions of the relationships between the variables of interest.

Its limitation notwithstanding, the current study showed that PTSSs may have mediating effects on the relationships between insecure attachment orientations and domains of quality of life. These findings might have relevant implications. Individuals who are exposed to potentially highly distressing conditions, such as low socioeconomic status [70] and chronic medical illness [71], or potentially traumatic events might benefit from psychoeducation programs focused on promoting favorable strategies for managing distress and coping with adverse experiences. These programs might be critical in preventing clinically relevant levels of PTSSs and poorer quality of life. Additionally, a careful evaluation of the role of insecure attachment orientations in managing distressing events might allow clinicians to design tailored interventions for individuals who report a poor quality of life. These interventions may be informed by various therapeutic approaches aimed at fostering the ability to process one’s bodily, cognitive, and behavioral posttraumatic reactions and enhancing self-regulation skills, such as trauma-focused mentalization-based treatment [72] or sensorimotor psychotherapy [73]. It is advisable for clinicians to help patients with insecure attachment orientations experience a sense of security [14] in the therapeutic relationship, avoiding enhancing their feelings of unworthiness and/or distrust towards others. This might be critical to assist them in recognizing the potential adverse consequences of the hyperactivation or deactivation of the attachment system on their daily functioning and adopting effective self- and inter-regulation strategies when facing distressing events. This might reduce the likelihood of suffering from PTSSs and improve their quality of life. 

## 5. Conclusions

The current study provides evidence on the potential pathways underlying the relationships between insecure attachment orientations and domains of quality of life, showing that PTSSs have mediating effects on these relationships. Accordingly, our findings suggest that insecure attachment orientations, which are closely linked to the hyperactivation or deactivation of the attachment system, might be related to poorer quality of life due to a heightened vulnerability for PTSSs in response to stressful or potentially traumatic events. Prevention programs and tailored clinical interventions focused on improving self- and inter-regulation strategies for coping with stressful or otherwise potentially traumatic events might enhance the quality of life across various areas of daily functioning in individuals with insecure attachment orientations. 

## Figures and Tables

**Table 1:** Descriptive statistics.

	Full Sample				
	(*n* = 497)				
	M	(SD)	Range	Skewness	Kurtosis
Age	32.48	(13.26)	18–65	0.87	−0.63
RQ–Attachment anxiety	−1.07	(4.85)	−12–12	0.13	−0.68
RQ–Attachment avoidance	0.4	(3.95)	−12–12	−0.09	−0.30
IES-R–Posttraumatic stress symptoms	42.09	(19.93)	0–87	−0.19	−0.70
WHOQOL-BREF–Physical health	14.46	(2.37)	8–20	−0.15	−0.54
WHOQOL-BREF–Psychological status	12.69	(2.65)	4.67–20	−0.05	−0.21
WHOQOL-BREF–Social relationships	13.67	(3.20)	4–20	−0.47	0.09
WHOQOL-BREF–Environment	13.11	(2.24)	6.5–19.5	0.04	−0.12

Note. RQ–Attachment anxiety = Relationship Questionnaire–Attachment anxiety scale; RQ–Attachment avoidance = Relationship Questionnaire–Attachment avoidance scale; IES-R–Posttraumatic stress symptoms = Impact of Event Scale-Revised–Posttraumatic stress symptoms scale; WHOQOL-BREF–Physical health = World Health Organization Quality of Life BREF–Quality of physical health scale; WHOQOL-BREF–Psychological status = World Health Organization Quality of Life BREF–Quality of psychological status scale; WHOQOL-BREF–Social relationships = World Health Organization Quality of Life BREF–Quality of social relationships scale; WHOQOL-BREF–Environment = World Health Organization Quality of Life BREF–Quality of environment scale.

**Table 2:** Sex differences.

	Males		Females		
	(*n* = 122)		(*n* = 375)		
	M	(SD)	M	(SD)	t_(495)_
Age	30.80	(12.83)	33.03	(13.36)	−1.62
RQ–Attachment anxiety	−1.81	(4.64)	−0.83	(4.89)	−1.95
RQ–Attachment avoidance	0.37	(3.89)	0.41	(3.97)	−0.09
IES-R–Posttraumatic stress symptoms	36.31	(20.37)	43.97	(19.44)	−3.74 **
WHOQOL-BREF–Physical health	14.83	(2.50)	14.33	(2.31)	2.03 *
WHOQOL-BREF–Psychological status	13.18	(2.88)	12.53	(2.56)	2.36 *
WHOQOL-BREF–Social relationships	13.33	(3.62)	13.79	(3.04)	−1.36
WHOQOL-BREF–Environment	13.73	(2.38)	12.91	(2.16)	3.56 **

Note. “Male” was coded as 1, and “Female” was coded as 2; RQ–Attachment anxiety = Relationship Questionnaire–Attachment anxiety scale; RQ–Attachment avoidance = Relationship Questionnaire–Attachment avoidance scale; IES-R–Posttraumatic stress symptoms = Impact of Event Scale-Revised–Posttraumatic stress symptoms scale; WHOQOL-BREF–Physical health = World Health Organization Quality of Life BREF–Quality of physical health scale; WHOQOL-BREF–Psychological status = World Health Organization Quality of Life BREF–Quality of psychological status scale; WHOQOL-BREF–Social relationships = World Health Organization Quality of Life BREF–Quality of social relationships scale; WHOQOL-BREF–Environment = World Health Organization Quality of Life BREF–Quality of environment scale; * *p* < 0.05; ** *p* < 0.001.

**Table 3:** Pearson’s *r* correlations between the investigated variables.

	2.	3.	4.	5.	6.	7.	8.
1. Age	−0.19 ***	−0.02	−0.24 ***	0.06	0.13 **	−0.01	−0.05
2. RQ–Attachment anxiety	–	0.08	0.25 ***	−0.30 ***	−0.40 ***	−0.42 ***	−0.25 ***
3. RQ–Attachment avoidance		–	0.10 *	−0.09 *	−0.13 **	−0.18 ***	−0.09 *
4. IES-R–Posttraumatic stress symptoms			–	−0.42 ***	−0.45 ***	−0.22 ***	−0.28 ***
5. WHOQOL-BREF–Physical health				–	0.67 ***	0.37 ***	0.52 ***
6. WHOQOL-BREF–Psychological status					–	0.53 ***	0.57 ***
7. WHOQOL-BREF–Social relationships						–	0.39 ***
8. WHOQOL-BREF–Environment							–

Note. RQ–Attachment anxiety = Relationship Questionnaire–Attachment anxiety scale; RQ–Attachment avoidance = Relationship Questionnaire–Attachment avoidance scale; IES-R–Posttraumatic stress symptoms = Impact of Event Scale—Revised–Posttraumatic stress symptoms scale; WHOQOL-BREF–Physical health = World Health Organization Quality of Life BREF–Quality of physical health scale; WHOQOL-BREF–Psychological status = World Health Organization Quality of Life BREF–Quality of psychological status scale; WHOQOL-BREF–Social relationships = World Health Organization Quality of Life BREF–Quality of social relationships scale; WHOQOL-BREF–Environment = World Health Organization Quality of Life BREF–Quality of environment scale; * *p* < 0.05; ** *p* < 0.01; *** *p* < 0.001.

**Table 4:** Regression models predicting domains of quality of life.

	β	SE	Partial *r*	t
**WHOQOL-BREF–Physical health**				
[Model: F(5,491) = 28.016, *p* < 0.001, R^2^ = 0.22]				
Sex	0.00	0.22	−0.01	−0.12
Age	−0.07	0.01	−0.07	−1.61
RQ—Attachment anxiety	−0.22 ***	0.02	−0.23	−5.22
RQ—Attachment avoidance	−0.04	0.02	−0.04	−0.89
IES-R—Posttraumatic stress symptoms	−0.38 ***	0.01	−0.37	−8.76
**WHOQOL-BREF–Psychological status**				
[Model: F(5,491) = 41.987, *p* < 0.001, R^2^ = 0.30]				
Sex	−0.02	0.24	−0.02	−0.40
Age	−0.02	0.01	−0.02	−0.52
RQ—Attachment anxiety	−0.31 ***	0.02	−0.33	−7.73
RQ—Attachment avoidance	−0.07	0.03	−0.08	−1.82
IES-R—Posttraumatic stress symptoms	−0.37 ***	0.01	−0.38	−9.15
**WHOQOL-BREF–Social relationships**				
[Model: F(5,491) = 30.556, *p* < 0.001, R^2^ = 0.24]				
Sex	0.13 **	0.30	0.15	3.30
Age	−0.14 **	0.01	−0.15	−3.45
RQ—Attachment anxiety	−0.41 ***	0.03	−0.41	−9.84
RQ—Attachment avoidance	−0.14 **	0.03	−0.15	−3.39
IES-R—Posttraumatic stress symptoms	−0.16 ***	0.01	−0.16	−3.69
**WHOQOL-BREF–Environment**				
[Model: F(5,491) = 16.564, *p* < 0.001, R^2^ = 0.14]				
Sex	−0.09 *	0.22	−0.09	−2.10
Age	−0.14 **	0.01	−0.14	−3.21
RQ—Attachment anxiety	−0.20 ***	0.02	−0.21	−4.63
RQ—Attachment avoidance	−0.05	0.02	−0.05	−1.21
IES-R—Posttraumatic stress symptoms	−0.24 ***	0.01	−0.24	−5.43

Note. “Male” was coded as 1, and “Female” was coded as 2; RQ–Attachment anxiety = Relationship Questionnaire–Attachment anxiety scale; RQ–Attachment avoidance = Relationship Questionnaire–Attachment avoidance scale; IES-R–Posttraumatic stress symptoms = Impact of Event Scale-Revised–Posttraumatic stress symptoms scale; WHOQOL-BREF–Physical health = World Health Organization Quality of Life BREF–Quality of physical health scale; WHOQOL-BREF–Psychological status = World Health Organization Quality of Life BREF–Quality of psychological status scale; WHOQOL-BREF–Social relationships = World Health Organization Quality of Life BREF–Quality of social relationships scale; WHOQOL-BREF–Environment = World Health Organization Quality of Life BREF–Quality of environment scale. * *p* < 0.05; ** *p* < 0.01, *** *p* < 0.001.

**Table 5:** Mediation models encompassing attachment anxiety as predictor.

	*B* *	95% CI	se	β
**WHOQOL-BREF–** **Physical health**				
[Total effect model: F(4,492) = 13.727, *p* < 0.001, R^2^ = 0.10]				
Predictor → Mediator	0.785	0.437, 1.133	0.177	0.191
Mediator → Outcome	−0.045	−0.055, −0.035	0.005	−0.375
Direct effect (Predictor → Outcome)	−0.106	−0.146, −0.066	0.020	−0.218
Total effect (Predictor → Outcome)	−0.141	−0.183, −0.099	0.021	−0.289
**WHOQOL-BREF–** **Psychological status**				
[Total effect model: F(4,492) = 27.016, *p* < 0.001, R^2^ = 0.18]				
Predictor → Mediator	0.785	0.437, 1.133	0.177	0.191
Mediator → Outcome	−0.050	−0.060, −0.039	0.005	−0.372
Direct effect (Predictor → Outcome)	−0.167	−0.210, −0.125	0.022	−0.306
Total effect (Predictor → Outcome)	−0.206	−0.251, −0.161	0.023	−0.377
**WHOQOL-BREF–** **Social relationships**				
Total effect model: F(4,492) = 33.914, *p* < 0.001, R^2^ = 0.22]				
Predictor → Mediator	0.785	0.437, 1.133	0.177	0.191
Mediator → Outcome	−0.025	−0.038, −0.012	0.007	−0.157
Direct effect (Predictor → Outcome)	−0.268	−0.322, −0.214	0.027	−0.406
Total effect (Predictor → Outcome)	−0.288	−0.341, −0.235	0.027	−0.436
**WHOQOL-BREF–** **Environment**				
[Total effect model: F(4,492) = 12.618, *p* < 0.001, R^2^ = 0.09]				
Predictor → Mediator	0.785	0.437, 1.133	0.177	0.191
Mediator → Outcome	−0.027	−0.037, −0.017	0.005	−0.244
Direct effect (Predictor → Outcome)	−0.094	−0.133, −0.054	0.020	−0.203
Total effect (Predictor → Outcome)	−0.115	−0.155, −0.075	0.020	−0.249

Note. WHOQOL-BREF–Physical health = World Health Organization Quality of Life BREF–Quality of physical health scale; WHOQOL-BREF–Psychological status = World Health Organization Quality of Life BREF–Quality of psychological status scale; WHOQOL-BREF–Social relationships = World Health Organization Quality of Life BREF–Quality of social relationships scale; WHOQOL-BREF–Environment = World Health Organization Quality of Life BREF–Quality of environment scale; Predictor = Relationship Questionnaire–Attachment anxiety scale; Mediator = Impact of Event Scale-Revised–Posttraumatic stress symptoms scale; * all estimates are significant at *p* < 0.001.

**Table 6:** Mediation models encompassing attachment avoidance as predictor.

	*B*	95% CI	se	β
**WHOQOL-BREF–** **Physical health**				
[Total effect model: F(4,492) = 13.727, *p* < 0.001, R^2^ = 0.10]				
Predictor → Mediatior	0.428 *	0.010, 0.845	0.212	0.085
Mediator → Outcome	−0.045 **	−0.055, −0.035	0.005	−0.375
Direct effect (Predictor → Outcome)	−0.021	−0.069, 0.026	0.024	−0.036
Total effect (Predictor → Outcome)	−0.040	−0.091, 0.010	0.026	−0.068
**WHOQOL-BREF–** **Psychological status**				
[Total effect model: F(4,492) = 27.016, *p* < 0.001, R^2^ = 0.18]				
Predictor → Mediator	0.428 *	0.010, 0.845	0.212	0.085
Mediator → Outcome	−0.050 **	−0.060, −0.039	0.005	−0.372
Direct effect (Predictor → Outcome)	−0.047	−0.097, 0.004	0.026	−0.069
Total effect (Predictor → Outcome)	−0.068 *	−0.122, −0.014	0.028	−0.101
**WHOQOL-BREF–** **Social relationships**				
[Total effect model: F(4,492) = 33.914, *p* < 0.001, R^2^ = 0.22]				
Predictor → Mediator	0.428 *	0.010, 0.845	0.212	0.085
Mediator → Outcome	−0.025 **	−0.038, −0.012	0.007	−0.157
Direct effect (Predictor → Outcome)	−0.109 **	−0.172, −0.046	0.032	−0.135
Total effect (Predictor → Outcome)	−0.120 **	−0.184, −0.056	0.032	−0.148
**WHOQOL-BREF–** **Environment**				
[Total effect model: F(4,492) = 12.618, *p* < 0.001, R^2^ = 0.09]				
Predictor → Mediator	0.428 *	0.010, 0.845	0.212	0.085
Mediator → Outcome	−0.027 **	−0.037, −0.017	0.005	−0.244
Direct effect (Predictor → Outcome)	−0.029	−0.076, 0.018	0.024	−0.051
Total effect (Predictor → Outcome)	−0.041	−0.089, 0.008	0.024	−0.071

Note. WHOQOL-BREF–Physical health = World Health Organization Quality of Life BREF–Quality of physical health scale; WHOQOL-BREF–Psychological status = World Health Organization Quality of Life BREF–Quality of psychological status scale; WHOQOL-BREF–Social relationships = World Health Organization Quality of Life BREF–Quality of social relationships scale; WHOQOL-BREF–Environment = World Health Organization Quality of Life BREF–Quality of environment scale; Predictor = Relationship Questionnaire–Attachment avoidance scale; Mediator = Impact of Event Scale—Revised–Posttraumatic stress symptoms scale; * *p* < 0.05; ** *p* < 0.001.

## Data Availability

The data presented in this study are available on request. The data are not publicly available due to GDPR 2016/79.
